# The best postoperative adjuvant therapy for patients with early stage cervical adenosquamous carcinoma

**DOI:** 10.1186/s12905-021-01588-8

**Published:** 2022-04-12

**Authors:** Yawen Liu, Haiyan Tu, Lingling Zhang, Meiling Zhong, Yanan Wang, Ling Li, Xiaojun Xiang

**Affiliations:** 1 Department of Oncology, Jiangxi Maternal and Child Health Hospital, No. 318 Bayi Road, Nanchang, 330006 China; 2 Department of Oncology, The First Affiliated Hospital of Nanchang University, No. 1227 Yongwaizheng Street, Donghu District, Nanchang, 330006 China

**Keywords:** Adenocarcinoma, Adenosquamous, Adjuvant treatment, Uterine cervical neoplasms, Radical hysterectomy, Radiotherapy

## Abstract

**Background:**

Cervical adenosquamous carcinoma (ASC) was previously thought to be a subtype of cervical adenocarcinoma, but recent studies have found that the clinical features of the two diseases are different. Moreover, the pathological characteristics, survival, prognosis, and optimal ASC therapy remain unknown. This study aims to retrospectively analyze the postoperative survival of patients with early-stage ASC and to evaluate their condition after treatment with postoperative concurrent chemoradiotherapy (CCRT) and prophylactic irradiation of the para-aortic lymphatic drainage area.

**Methods:**

This study enrolled 131 patients with pathologically confirmed ASC screened from 3502 patients with confirmed stage I–II cervical cancer diagnosis who had completed surgical treatments in our hospital. Among the 131 enrolled patients, 75 patients received CCRT, 33 patients received chemotherapy (CT), and 23 patients did not receive adjuvant treatment (named surgery alone (S alone). Of the 75 patients CCRT, 43 patients received prophylactic irradiation of the para-aortic lymphatic drainage area. The efficacy of the postoperative treatments of patients among groups (CCRT, CT, and S alone) was compared.

**Results:**

The median follow-up time, age, and overall survival (OS) were 76 months, 43 years, and 74 months, respectively. The 3- and 5-year survival rates were 82% and 71.4%, respectively. The median disease-free survival (DFS) was 64 months. Cox regression analysis showed that postoperative adjuvant treatment modalities and positive lymph node metastases were associated with OS and DFS. Patients who received CCRT treatment had higher OS and DFS than those with CT and S alone. Prophylactic irradiation of the para-aortic lymphatic drainage area did not improve the OS and DFS of patients with CCRT treatment. However, further subgroup analysis suggested that it might improve survival rates in patients who had positive pelvic lymph nodes as confirmed by postoperative pathology.

**Conclusion:**

Postoperative CCRT improved the survival rates in patients with early-stage ASC. The value of prophylactic irradiation of the para-aortic lymphatic drainage area remains debatable, but it may benefit patients with pelvic lymph node involvement.

## Background

Cervical cancer is a common gynecological malignancy, and contributes to 270,000 deaths annually [1]. Cervical cancer can be affected by multiple factors, such as human papillomavirus (HPV) infection, marriage at an early age, multiple childbirths, multiple sexual partners, and genetic factors. With cervical screening and the use of vaccines, cervical cancer can be prevented and controlled in a timely manner. The management of early-stage cancer is vital for improving patient prognosis and survival.

Cervical adenosquamous carcinoma (ASC) is a special histological type of cervical cancer with an extremely low incidence rate, accounting for only 5–10% of cervical cancer cases [2, 3]. The prognosis of ASC was first reported in 1956, and its formation was suggested to result from the concurrent differentiation and development of reserve cells into glandular and squamous cells, resulting in tumoral tissues with components of both adenocarcinoma and squamous cell carcinoma [4, 5]. Previous studies have suggested that ASC is a subtype of cervical adenocarcinoma. Since they have always been studied together as a whole [6, 7], no difference in outcomes between the two carcinomas has been described [8–11]. However, recent studies have demonstrated that the clinical characteristics of the two carcinomas are not the same. ASC exhibits a higher invasiveness and probability of lymph node metastasis, resulting in a worse prognosis than cervical adenocarcinoma [12–14]. One study has suggested that para-aortic lymph node metastasis is a major cause of cervical cancer relapse and that the uncontrolled rate of para-aortic lymph node involvement after routine concurrent chemoradiotherapy (CCRT) is 10–25% [15]. Therefore, it is believed that relapse in the para-aortic lymph node area may be a crucial factor affecting the prognosis of patients with cervical cancer. Although there are no standard principles for the treatment of this special pathological type of cervical cancer, the standard guidelines and criteria for the diagnosis and therapy of cervical cancer are often followed. Thus, the pathological characteristics, survival, prognosis, and optimal therapy for ASC remain unknown.

The present study aims to retrospectively analyze the postoperative survival of patients with early-stage ASC, investigate the appropriate postoperative adjuvant treatment modalities, and explore whether prophylactic irradiation of the para-aortic lymphatic drainage area affects survival. 

## Methods

### Participants and data collection

This retrospective study screened 3502 patients with early-stage cervical cancer (International Federation of Gynecology and Obstetrics [FIGO]2009 stage IB1-IIA2) who were admitted to Jiangxi Maternal and Child Health Hospital between January 2005 and September 2016. Patients with ASC, as confirmed by postoperative pathology, were enrolled in this study. Exclusions included other histological types, failure to undergo surgery, cervical conization, preoperative neoadjuvant chemotherapy (CT), postoperative adjuvant therapy in other medical centers, brachytherapy, a second primary cancer, and incomplete data. Finally, 131 patients were enrolled. The clinicopathologic characteristics, treatment modalities, and relapse patterns of the participants were then recorded. The protocols and contents of this research were approved by the ethical board of the Institute of the Jiangxi Maternal and Child Health Hospital. Written informed consent to participate in the study was obtained from all the patients.

### Treatment methods

Of the 131 patients enrolled in the study, 120 patients had undergone the radical hysterectomy, 6 patients had the radical trachelectomy, 4 patients received total laparoscopic hysterectomy, and 1 had the patient received lesion resection. Based on surgical exploration and postoperative pathology, the high-risk factors were identified as positive parametrium, surgical margin, and lymph nodes, and the intermediate-risk factors as tumor diameter ≥ 4 cm, stromal invasion ≥ 1/2, and presence of lymphovascular space invasion. Patients with a high-risk factor or more than two intermediate-risk factors were treated with CCRT. Patients with the intermediate-risk factor vessel carcinoma embolus received chemotherapy alone or observation, and those with the intermediate-risk factor interstitial infiltration > 1/2 received pelvic radiotherapy or observation (no patients in this study chose radiotherapy). For patients with an intermediate risk factor, treatment was chosen according to the individual wishes of each patient. Patients who received radiotherapy met the Sedlis criteria as revealed by their postoperative pathology.

We used a Sweden Elekta linear accelerator for 6-MV X-rays with two-field irradiation or intensity modulated radiation therapy. The target volume included the vaginal stump, parametrium, and pelvic lymph node drainage area (common iliac, internal and external iliac, presacral, and obturator lymph nodes). For patients who have undergone radical resection, the status of para-aortic lymph nodes was determined according to the pathological results of intraoperative abdominal para-aortic lymph node dissection or biopsy and imaging features of initial diagnosis. If one of the results indicated that the para-aortic lymph node was positive, that lymph nodes was considered positive. Patients with positive common iliac or para-aortic lymph nodes received para-aortic extended-field irradiation. In this study, prophylactic para-aortic irradiation was performed in 43 patients without metastasis in the common iliac and para-aortic lymph nodes as shown by postoperative pathology and imaging (upper boundary: lower border of the left renal vein). The radiotherapy dose was 45 Gy, 1.8 Gy/session, for a total of 25 sessions. One patient with bulky pelvic lymph node metastases underwent a laparoscopic hysterectomy. Since the surgeons were not confident that the lesion was completely removed, they placed a titanium clip and suggested that the patient undergo a postoperative radiation boost. We administered a dose of radiotherapy of up to 60 Gy in the marked area, and postoperative magnetic resonance imaging did not indicate any obvious residual disease.

A total of 75 patients received concurrent CT. Among them, 32 received paclitaxel liposome (90 mg) and nedaplatin (30–35 mg/m^2^), 36 patients received docetaxel (25–30 mg/m^2^) + carboplatin (area under the plasma concentration time curve [AUC] = 2), 3 patients received a single dose of nedaplatin (35 mg/m^2^), and 4 patients received carboplatin (AUC = 3) on a weekly cycle. A total of 33 patients received adjuvant CT alone. Among them, 23 received docetaxel (75 mg/m^2^) + carboplatin (AUC = 5) and 10 patients received paclitaxel liposome (2 with 135 mg/m^2^ and 8 with 175 mg/m^2^) + nedaplatin (80 mg/m^2^), on a 3-week cycle for a total of 4 cycles. See Table 1.

**Table 1:** Concurrent chemoradiotherapy (CCRT) for patients with cervical adenosquamous carcinoma

	CCRT (n = 75)	CT (n = 33)
Paclitaxel liposome + nedaplatin	32	10
Docetaxel + carboplatin	36	23
Nedaplatin	3	0
Carboplatin	4	0

*CT* chemotherapy

### Follow-up

The patients were followed up once every 3 months in post-treatment years 1–2, once every 6 months in years 3–5, and once every year thereafter. Medical records were reviewed for regular follow-ups, and telephone calls were made to patients for non-scheduled follow-ups. The data of patients lost to follow-up were censored from the date of loss to follow-up.

### Statistical methods

Statistical analysis was performed using the SPSS software version 19.0 (SPSS Inc., Chicago, IL, USA). The χ^2^ test, Kaplan–Meier method, and log-rank test were used for counting data, calculating the survival rates, and performing the intergroup comparisons. The statistical significance level was set at *p* < 0.05.

## Results

### Participant characteristics

A total of 3502 patients with early-stage cervical cancer (FIGO2009 stage IB1–IIA2) were included in this study. Among them, 141 patients with ASC were selected. Ten of the selected patients were excluded due to incomplete data or not receiving postoperative treatments in our hospital. Finally, 131 patients were enrolled. The median age of the participants was 43 years (range: 22–76 years). Of the enrolled patients, 98, 18, 13, and 2 participants had stage IB1, IB2, IIA1, and IIA2 cancer, respectively (baseline data are shown in Table 2).

**Table 2:** Clinicopathologic characteristics of 131 patients

Mean age	43 (22 − 76) years	
Characteristics	Number of cases	Percentage (%)
FIGO stage		
IB1	98	74.8
IB2	18	13.7
IIA1	13	9.9
IIA2	2	1.5
Tumor size		
< 4 cm	48	36.6
> 4 cm	83	63.4
DSI		
< 1/2	39	29.8
> 1/2	92	70.2
LVSI		
Yes	50	38.2
No	81	61.0
LN metastasis		
Yes	57	43.5
No	74	56.6
Surgical procedures		
Radical trachelectomy	6	4.6
Radical hysterectomy	120	91.6
Laparoscopic hysterectomy	4	3.1
Local resection	1	0.7
Adjuvant therapy		
No (S alone)	23	17.5
CCRT	75	57.3
CT	33	25.2
Prophylactic para-aortic irradiation		
Yes	43	32.8
No	88	67.2
Relapse pattern		
Distant metastasis	18	13.7
Local recurrence	8	6.1

*FIGO* International Federation of Obstetrics and Gynecology; *LVSI* lymphovascular space invasion; *DSI* depth of cervical stromal invasion; *LN* lymph node; *S* surgery; *CCRT* concurrent chemoradiotherapy; *CT* chemotherapy

### Survival time and relapse

**Fig. 1:**
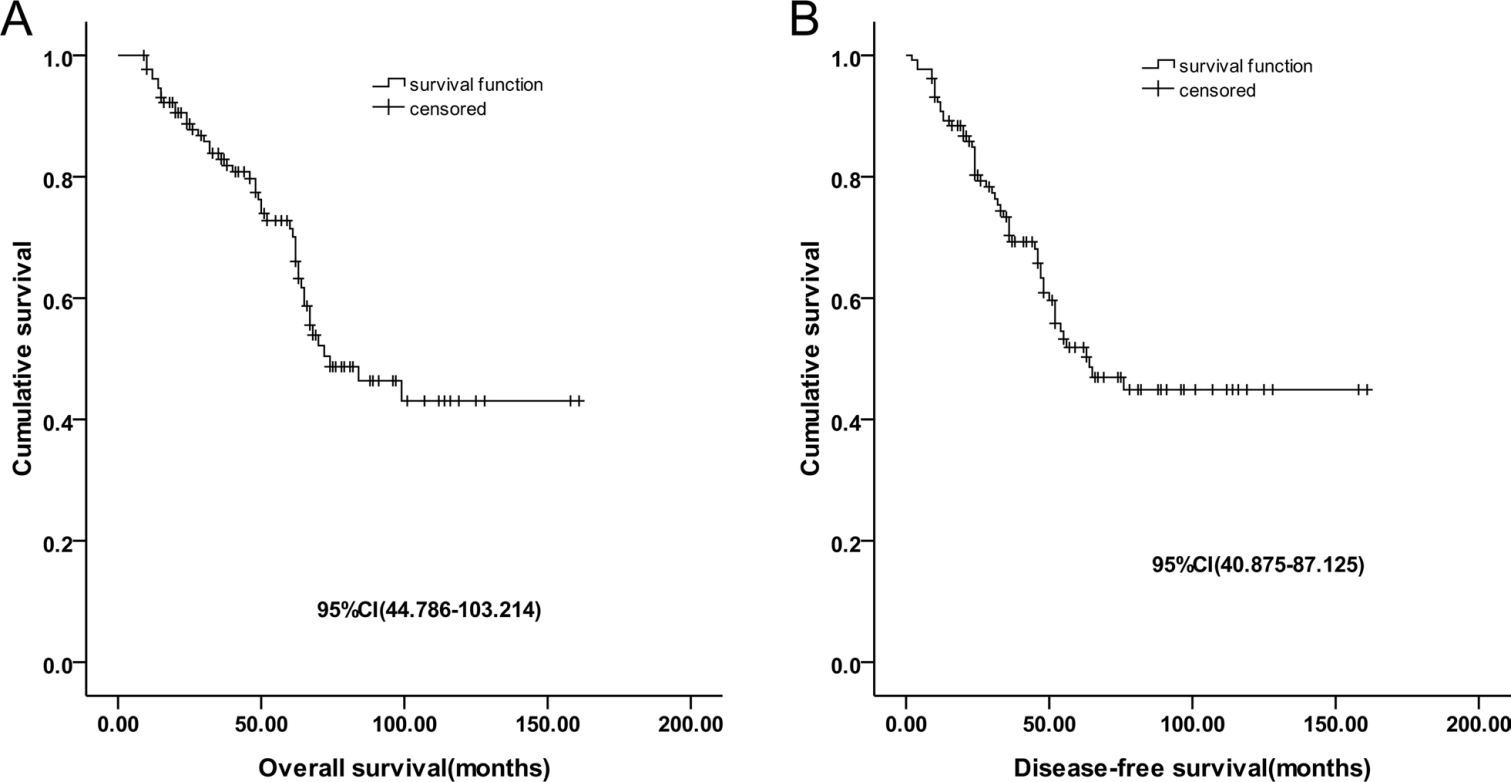
**A** Overall survival curve of 131 patients. **B** Disease-free survival curve of 131 patients

The median follow-up period and overall survival (OS) were 76 months (range: 4–156 months) and 74 months (95% CI: 44.79–103.21 months), respectively, while the 3-year and 5-year survival rates were 82% and 71.4%, respectively. The disease-free survival (DFS) was 64 months (95% CI: 40.88–87.13 months) and the 3-year and 5-year DFS rates were 69.8% and 51%, respectively. There were 26 cases of relapse (8 of local recurrence and 18 cases of distant metastasis). The cases of distant metastasis included 9 cases of lung metastasis, 4 of bone metastasis, 1 liver metastasis, 2 distant metastasis and 2 local recurrence, and distant metastasis at unknown sites (Fig. 1A, B).

### Analysis of survival-related factors

The clinicopathological factors, such as FIGO staging, postoperative adjuvant therapy methods, lymphovascular space invasion, depth of cervical stromal invasion; tumor diameter, lymph node metastasis, and prophylactic irradiation of the para-aortic lymphatic drainage area were grouped, and their impacts on survival were analyzed. The Cox regression analysis results suggested that lymphovascular space invasion was only associated with DFS and did not appear to be related to OS (Table 3). Moreover, the results also indicated that postoperative adjuvant treatment modalities (95% CI: 1.530–2.592, *p* < 0.001 and 95% CI: 1.825–3.430, *p* < 0.001, respectively) and lymph node metastasis (95% CI: 0.150–0.584, *p* < 0.001 and 95% CI: 0.182–0.737, *p* = 0.050 respectively) were related to OS and DFS, respectively. (Table 3).

**Table 3:** Cox regression analyses for factors predicting overall survival and disease-free survival

	Overall survival			Disease-free survival		
	Wald	*p*	95%CI	Wald	*p*	95%CI
FIGO stage	0.441	0.507	0.805–2.165	2.247	0.134	0.897–2.262
Treatment modalities	26.223	0.000	1.530–2.592	32.476	0.000	1.825–3.430
LVSI	3.396	0.065	0.270–1.041	3.875	0.049	0.233–0.997
Prophylaxis irradiation	3.614	0.057	0.291–1.019	0.973	0.324	0.379–1.378
DSI	0.941	0.332	0.697–2.912	3.806	0.051	0.996–5.167
LN metastasis	12.367	0.000	0.150–0.584	7.929	0.050	0.182–0.737
Tumor size	0.169	0.681	0.605–1.388	0.018	0.893	0.621–1.514

*FIGO* International Federation of Obstetrics and Gynecology, *LVSI* lymphovascular space invasion, *DSI* depth of cervical stromal invasion, *LN* lymph node

**Table 4:** Summary of the survival rate for patients with early-stage ASC in recent years

Study	Cell type	FIGO	5-y OS	5-y DFS
Yasuda et al. [2]	ASC (n = 28) AC (n = 81)	IB1	82.4% 92.4%	82% 92%
Barquet et al. [19]	ASC (n = 14) AC (n = 57)	IA2-IIA1	100% 97.8%	92.3% 98.1%
Mabuchi et al. [11]	ASC (n = 20) AC (n = 143)	IA2-IIB	–	79.2% 74.1%
Beak et al. [20]	ASC (n = 72) AC (n = 265)	IA2-II	88% 92%	85% 88%
Twu et al. [21]	ASC (n = 321) AC (n = 811)	I-II	94.2% (low risk) 65.8% (high risk)	88.6% (low risk) 55.1% (high risk)
Current study	ASC (n = 131)	IB1-IIA2	71.4%	51%

*FIGO* International Federation of Obstetrics and Gynecology; *AC* adenocarcinoma; *ASC* adenosquamous carcinoma; *5-y OS* 5-year overall survival; *5-y DFS* 5-year disease-free survival

### Survival following postoperative adjuvant therapy

**Fig. 2:**
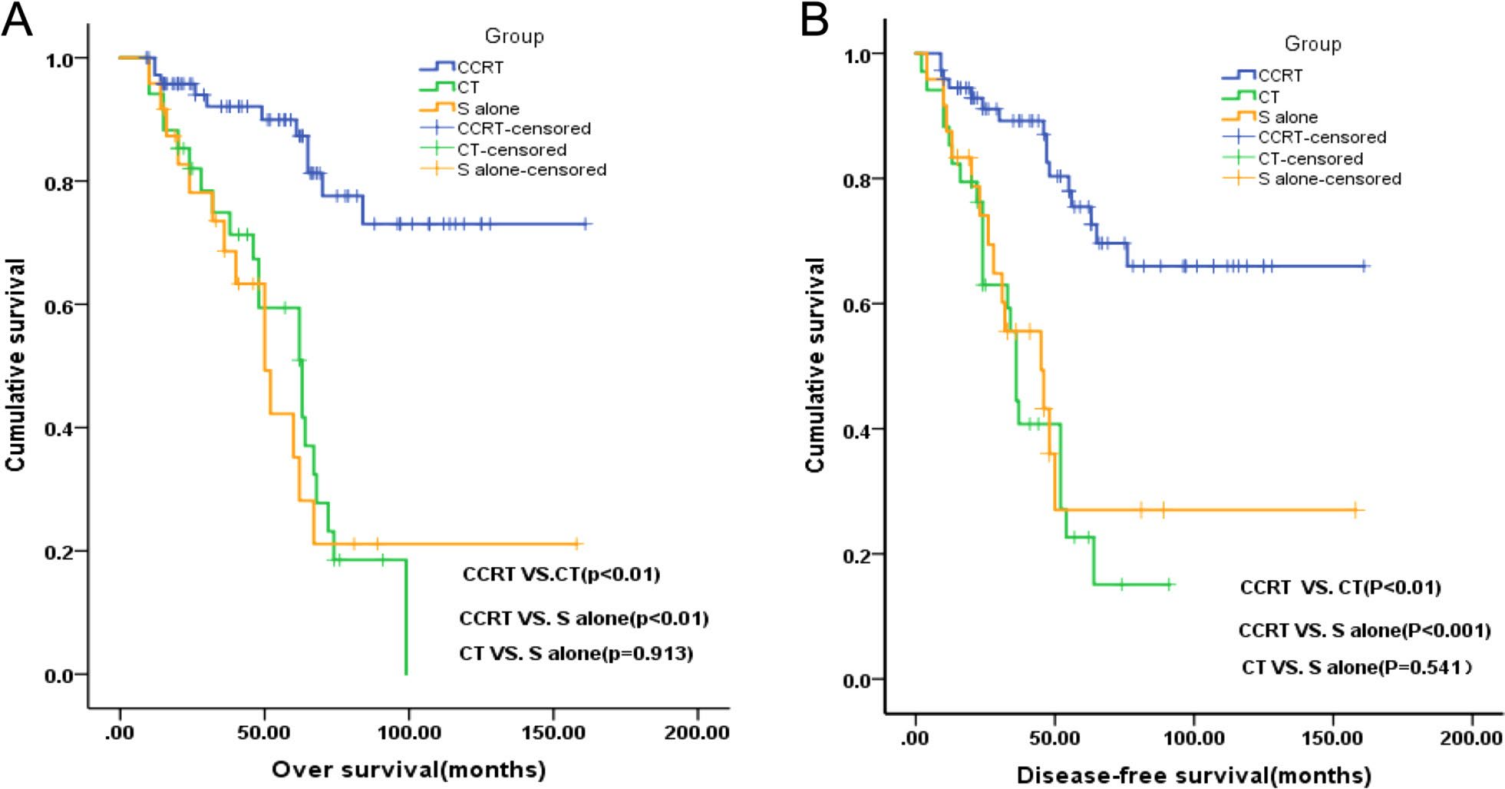
**A** Overall survival curves of patients who received the three postoperative adjuvant therapy methods. **B** Disease-free survival curves of patients who received the three postoperative adjuvant therapy methods. Group 1, received concurrent chemoradiotherapy (CCRT); Group 2, only received chemotherapy (CT); and Group 3, received no therapy after surgery (S alone)

We compared the postoperative adjuvant therapy methods (CCRT, CT and surgery[S] alone). The results demonstrated that, in terms of OS, CCRT (n = 75) was significantly superior to S alone (n = 23) (χ2 = 17.719, *p* < 0.001) and to CT (n = 33) (χ2 = 23.584, *p* < 0.001), but there was no difference between the patients who received CT or underwent S alone (χ2 = 0.012, *p* = 0.913). Similarly, for DFS, CCRT was significantly better than S alone (χ2 = 12.388, *p* < 0.001) and CT χ^2^ = 24.438, *p* < 0.001) on DFS, but there was no difference between the patients who received CT or underwent S alone (χ^2^ = 0.373, *p* = 0.541) (Fig. 2A, B).

### Survival following prophylactic para-aortic irradiation

**Fig. 3:**
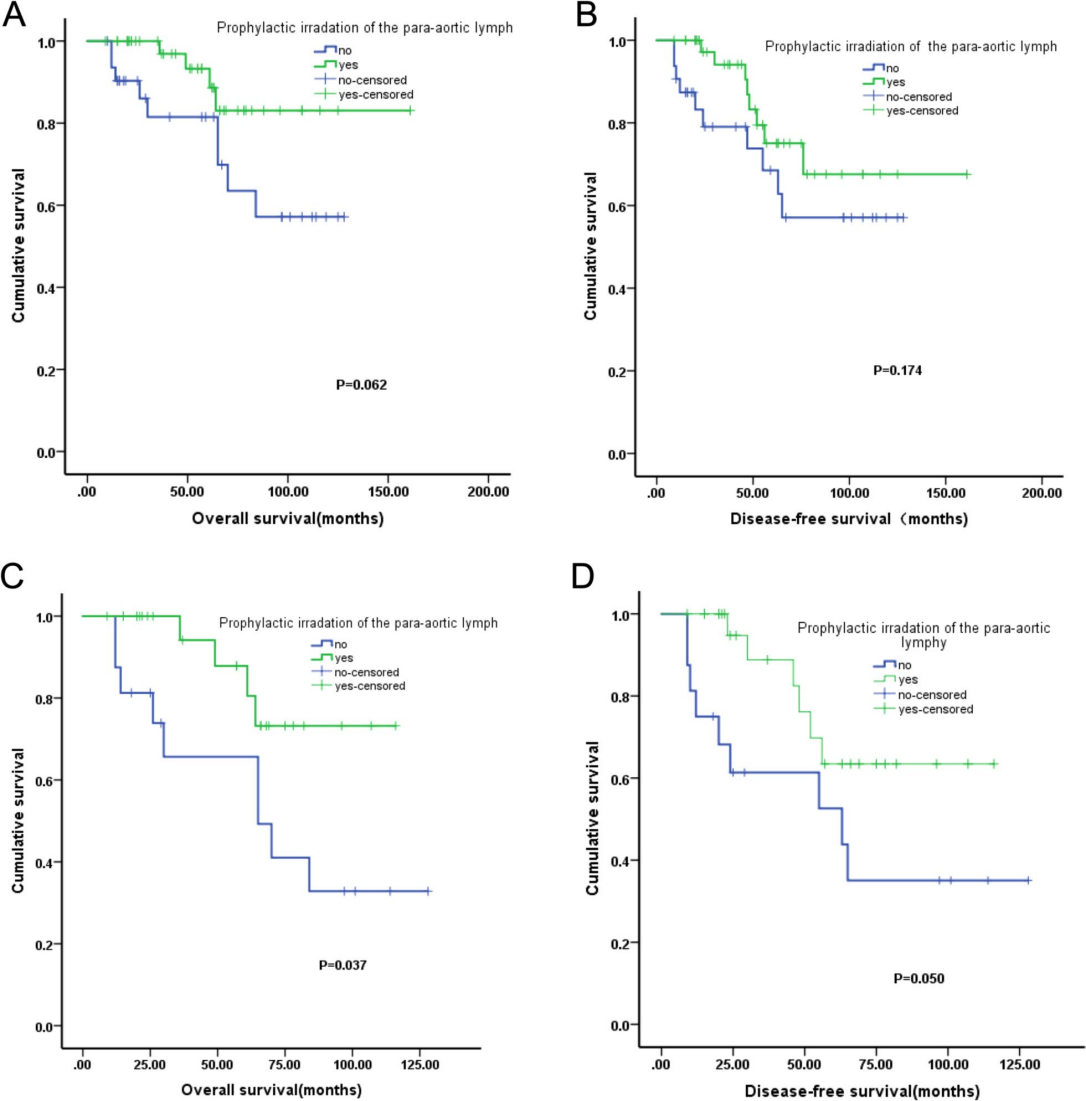
**A** Overall survival curves of patients who received prophylactic para-aortic irradiation. **B** Disease-free survival curves of patients who received prophylactic para-aortic irradiation. **C** Results of subgroup analysis of the positive pelvic lymph nodes. Overall survival rate curves of patients with positive lymph nodes with or without prophylactic irradiation of the para-aortic lymphatic drainage area. **D** Results of subgroup analysis of the positive pelvic lymph nodes. Disease-free survival rate curves of patients with positive lymph nodes with or without prophylactic irradiation of the para-aortic lymphatic drainage area

In this study, 75 patients received CCRT. Among these, 43 patients received prophylactic para-aortic irradiation, and 32 patients did not receive prophylactic para-aortic irradiation. There was no statistical difference in improvement of OS between patients who did and did not receive prophylactic para-aortic irradiation (χ^2^ = 3.483; *p* = 0.062). Additionally, there was no significant difference in DFS between the patients with and without prophylactic para-aortic irradiation (χ^2^ = 1.845; *p* = 0.174) (Fig. 3A, B).

### Results of the subgroup analysis

Overall, 57 patients were found to have positive nodes after undergoing a radical hysterectomy. Among these, 42, 10, and 5 patients received CCRT, CT and S alone respectively. The pelvic lymph node positivity was used as a stratification factor for subgroup analysis. The results indicated that the survival rate was significantly higher in patients who received prophylactic irradiation of the para-aortic lymphatic drainage area (n = 26) than in those patients who had not received prophylactic irradiation (n = 16) (χ^2^ = 4.350, *p* = 0.037). But there was no no significant difference in disease-free survival between these two patient groups (χ^2^ = 3.843, *p* = 0.050) (Fig. 3C, D).

## Discussion

Cervical cancer is one of the most common malignancies among women worldwide. While this disease’s morbidity and mortality has significantly decreased due to the popularity of cervical screening [16], because screening is not widely available worldwide, cervical cancer continues to be a threat to patient health, especially in low-income and middle-income territories [1]. ASC, a rare subtype of cervical cancer, has been found to have a poor prognosis due to its high degrees of invasiveness and malignancy; therefore, the reasonable management of ASC is vital. The present study retrospectively analyzed the effects of postoperative management and adjuvant therapy on the outcomes of ASC patients.

With the development of diagnosis and treatment technology, the management of early cervical cancer is becoming increasingly effective. For example, screening procedures, especially when combined with HPV DNA tests, provide an opportunity to identify pre-cancerous lesions and increases the timely diagnosis of early cervical cancer [17]. Moreover, the detection accuracy of lymph node metastasis in early cervical cancer has been greatly improved [18]. However, no large-scale prospective study has explored this special pathological type of cervical cancer to date, because of the low incidence of cervical adenosquamous carcinoma. Most retrospective studies have conducted investigations that included cervical adenocarcinoma. Many studies have investigated the survival and prognostic significance of patients with early-stage ASC and cervical adenocarcinoma. We summarized some of these in Table 4, showing 65.8% to 100% of 5-year OS rates and 55.1% to 92.3% of 5-year DFS rates [2, 11, 19–21]. Our study showed that the median OS was 74 months, and the 3-and 5-year OS rates were 82% and 71.4%, respectively. The median DFS was 64 months, and the 3- and 5-year DFS rates were 69.8% and 51%, respectively. The survival rates of the patients in our study were lower than those in previous studies, which may be explained by the following: first, most of the previous studies had few cases of ASC, and second, the short follow-up time in our study may have led to statistical bias.

The treatment of cervical cancer is dictated the by FIGO staging system; in this system, the appropriate surgical and postoperative radiotherapy and chemotherapy approaches are recommended for patients with early-stage diseases. For patients with early-stage cervical cancer, the primary treatment choices are radical hysterectomy and pelvic lymphadenectomy, which are performed using minimally invasive surgical techniques (laparoscopy or robotics) [22, 23]. However, for patients of childbearing age, preservation of reproductive function is desirable [23]. Of the 131 patients with cervical ASC in our study, 124 patients (94.7%) underwent radical hysterectomy, 6 patients (4.6%) underwent fertility-preserving cervical canal resection, and 1 patient (0.7%) underwent resection of the lesion only.

Results of Cox regression analysis in our study indicated that postoperative CCRT is associated with survival, although, the therapeutic effectiveness of primary chemoradiation for cervical adenocarcinoma cancer and ASC is still unclear. Peter et al. reported that adenocarcinoma and ASC of the cervix are associated with worse OS when treated with radiation alone, but have similar progression free and overall survival compared to squamous cell carcinomas of the cervix when treated with cisplatin-based chemoradiation [24]. Lee et al. concluded that intermediate/high-risk patients with ASC may be successfully treated with postoperative CCRT [25]. In both studies, ASC was compared to squamous and adenocarcinoma rather than to itself, which may lead to bias.

Moreover, our study showed that 43 patients who received prophylactic para-aortic irradiation did not have improved survival rates. Although several studies have investigated the efficacy of prophylactic para-aortic irradiation for advanced stage cervical cancer [26–31], the value of prophylactic para-aortic irradiation remains controversial. Some studies claimed that prophylactic para-aortic irradiation improves survival and reduces the risk of recurrence [26–28], whereas the results from other studies supported contrary claims: prophylactic para-aortic irradiation does not reduce the risk of recurrence for patients with advanced-stage cervical carcinoma [29–31]. No randomized controlled study has explored the value of prophylactic para-aortic irradiation for treating ASC. In fact, our study is the first and largest sample to analyze the effects of different irradiation treatment modalities on survival.

Previous studies have found that FIGO stage, tumor diameter, lymphovascular space invasion and lymph node metastasis are independent prognostic factors for poor survival in patients with early-stage cervical cancer [11, 20, 32–34]. In the results of our study, lymph node metastasis rather than tumor diameter or depth of cervical stromal invasion was the independent prognostic factor for ASC. It is worth noting that in our study, pelvic lymph node metastasis status was used as a stratification factor for analysis, and the results demonstrated that the prophylactic irradiation of the para-aortic lymphatic drainage area improved survival rates in patients with positive pelvic lymph nodes. Interestingly, this is also the first study that specifically analyzed the impact of this radiotherapy modality on survival, which can serve as a reference in the clinical setting.

Overall, our study only included patients with early-stage ASC who had undergone surgery. Specifically, 130 of the 131 included patients received the standard surgery for cervical cancer. Moreover, the postoperative adjuvant therapy method was relatively standardized, as the chemotherapy regimen, irradiation, and radiotherapy were completed in the same unit. Thus, we ensured the therapy regimens’ consistency and excluded the interference of subsequent therapies. Nevertheless, our study has several limitations. First, we did not directly compare ASC, adenocarcinoma, and squamous cell carcinoma. Second, patients who received prophylactic para-aortic irradiation were not randomized, and most received surgeon’s recommendations extended-field irradiation based on their intraoperative findings. Third, the proportion of patients enrolled in this study before 2010 was low, and the role of CCRT in cervical cancer treatment has yet to be confirmed. Multicenter, randomized, large-sample prospective studies should be conducted to assess the efficacy of concurrent para-aortic irradiation for ASC.

## Conclusion

In summary, the incidence of ASC was extremely low and postoperative adjuvant therapy and lymph node metastasis were independent prognostic factors in patients with early-stage ASC who underwent surgery. Furthermore, patient prognosis was improved by postoperative CCRT, which is likely to become a standard therapy. Prophylactic irradiation of the para-aortic lymphatic drainage area can reduce relapse in patients with positive pelvic lymph nodes. The data presented herein could potentially provide new perspectives on clinical treatment.

## Data Availability

The analyzed data sets generated during the study are available from the corresponding author on reasonable request.

## Abbreviations

HPV: Human papillomavirus
ASC: Adenosquamous carcinoma
CCRT: Concurrent chemoradiotherapy
CT: Chemotherapy
AUC: Area under the plasma concentration time curve
OS: Overall survival
DFS: Disease-free survival
